# Serum levels of angiotensin-converting enzyme 2 in children with Kawasaki disease

**DOI:** 10.1007/s10238-022-00933-x

**Published:** 2022-11-07

**Authors:** Yi Gan, Yawei Feng, Xiaoqin Zhou, Heng Li, Guirong Wang, Maidina Aini, Junhua Shu, Danna Tu

**Affiliations:** Department of Pediatric, Maternal and Child Health Hospital of Hubei Province, Tongji Medical College, Huazhong University of Science and Technology, No.745 Wu Luo Road, Hongshan District, Wuhan City, Hubei Province People’s Republic of China

**Keywords:** Kawasaki disease, ACE2 levels, Coronary artery damage, Intravenous immunoglobulin treatment, Mucocutaneous lymph node syndrome

## Abstract

Kawasaki disease (KD) has replaced rheumatic fever as the main cause of acquired heart disease in Japanese, American, and Chinese children. Polymorphisms in angiotensin-converting enzyme may be associated with susceptibility to KD, but the association of angiotensin-converting enzyme 2 (ACE2) with vascular endothelial injury in KD and the possibility for prognosis of vascular injury in KD by evaluating changes in serum ACE2 have not yet been assessed. Thus, this study aimed to investigate ACE2 levels in patients with KD to further explore the relationship between ACE2 and vascular injury in KD. Blood samples were collected from 49 children with KD before intravenous immunoglobulin treatment and 28 healthy children in the same period as the control group. Clinical data were collected from the patients and serum ACE2 levels of all participants were measured using an enzyme-linked immunosorbent assay. Serum ACE2 levels were significantly higher in the KD group than in the control group, and were negatively correlated with platelet levels in patients with KD. Serum ACE2 levels are related to the pathogenesis of KD and may be used as a potential serum marker for KD diagnosis.

## Introduction

KD is a systemic vasculitic disease in children under 5 years of age. Clinically known as mucocutaneous lymph node syndrome, which is typically characterized by persistent fever (for more than 5 days), diffuse inflammation of the mucosa (such as dry red rhagadia of the lips and bayberry tongue), non-suppurative conjunctivitis, large non-suppurative cervical lymph nodes (> 1.5 cm in diameter), polymorphic rash, and angioneurotic edema of the extremities [1, 2]. Approximately 25% of untreated children with KD experience coronary artery lesions (CAL), and KD has replaced rheumatic fever as the main cause of acquired heart disease in children in Japan, the United States, and China. Studies have demonstrated that damage to the vascular endothelium occurs in KD [3, 4].

ACE2, a congenic compound of the angiotensin-converting enzyme, is expressed on all endothelial cells and also ubiquitously expressed in cardiomyocytes, cardiac fibroblasts, and coronary endothelial cells [5]. Studies have shown that ACE2 and Ang1-7 in vascular endothelium can protect endothelial cell function and can regulate vascular function by regulating the release of nitric oxide and oxidative stress [6]. Local overexpression of ACE2 improves endothelial function and significantly inhibits the development of early atherosclerosis [7]. In addition, it has been shown that overexpression of ACE2 in endothelial progenitor cells (EPC) can improve their function by inhibiting apoptosis and oxidative stress [8]. ACE, another M2 family protein belonging to the same metalloprotease family as ACE2, has shown reduced expression in children with KD and significantly increased activity after the application of intravenous immunoglobulin (IVIG) therapy [9]. Therefore, we speculated that ACE2 may also be associated with vascular endothelial injury in KD. Once the vascular endothelial injury occurs, ACE2 can be released into the serum, and the prognosis of vascular injury in KD may be evaluated by detecting ACE2 changes in serum.

However, the relationship between ACE2 and KD has not yet been studied. Hence, our research aimed to investigate the relationship between serum ACE2 levels and coronary artery injury in patients with KD.

## Methods

### Patients

Patients were included in the study according to KD diagnosis based on diagnostic criteria from the American Heart Association’s 2017 KD guidelines [10]. A total of 49 children (30 boys and 19 girls, mean age: 32 months) with acute febrile KD at the Maternal and Child Health Hospital of Hubei Province were selected, and 28 children of similar age with normal physical examinations (15 boys and 13 girls, mean age: 25 months) were selected as the control group. Children with other immune (such as systemiconset juvenile idiopathic arthritis and systemic lupus erythematosus), metabolic diseases, hematological diseases, severe liver and kidney diseases, and other heart diseases were excluded from this study. Blood samples were collected from all patients before and after IVIG therapy, and echocardiographic parameter results were obtained one day before IVIG treatment or within 2 weeks of onset of KD. Patients with *Z* values < 2 were included in the KD-no cardiac artery lesions (KD-nCAL) group, those with *Z* values > 2 were included in the KD-cardiac artery lesions (KD-CAL) group, and those with *Z* values > 2.5 were included in the KD with coronary artery aneurysm (KD-CAAs) group [11, 12]. The Lambda-Mu-Sigma method proposed by T Kobayashi were used for *Z*-score [13]. Informed consent was obtained from the guardians of each participant, and the study was approved by the Medical Ethics Committee of the Maternal and Child Health Hospital of Hubei Province.

### Sample collection and processing

Blood samples from patients with KD were collected before and after IVIG treatment, while blood samples from the healthy control group were collected during routine health visits. The collected venous whole blood samples were centrifuged at 1500 rpm for 5 min, and the blood serum was collected, labeled, aliquoted, and stored at − 80 °C until testing.

### Measurement of serum ACE2 concentrations and clinical parameters

ACE2 serum concentrations were measured in all participants using Human ACE2 DuoSet ELISA kits (DY933-05, R&D, USA) according to the manufacturer's instructions. The assay range of the ELISA Kit is 0.3–20 ng/ml. All samples were analyzed in duplicate. Clinical parameters, including white blood cell count (WBC), red blood cell count (RBC), hemoglobin (Hb), Plt, neutrophil count (N), lymphocyte count (L), C-reactive protein (CRP), erythrocyte sedimentation rate (ESR), aspartate aminotransferase (AST), alanine aminotransferase (ALT), procalcitonin (PCT) and creatine kinase-MB (CK-MB), were also collected.

### Statistical analysis

According to the Kolmogorov–Smirnov test, the data from this study conformed to a normal Gaussian distribution. All data are shown as mean ± standard error of mean (SEM) or number. Differences between the groups were assessed using an unpaired 2-tailed t-test. All statistical analyses were performed using SPSS software (version 21.0; SPSS, Inc., Chicago, IL, USA). Statistical significance was set at *p* < 0.05.

## Results

There was no significant difference in age between the healthy control group (25.77 ± 9.72 months) and the KD group (32.78 ± 24.97 months) (*P* = 0.235). In 49 patients with KD, 43 cases were complete KD, 6 cases were incomplete KD. All KD patients were divided into the KD-CAL group (n = 22, *Z* > 2) and KD-nCAL group (*n* = 27, *Z* < 2), which was then further divided into the KD-CAA group (*n* = 16, *Z* > 2.5) and KD-nCAA group (*n* = 33, *Z* < 2.5).

### Serum ACE2 levels in children with KD

**Fig. 1:**
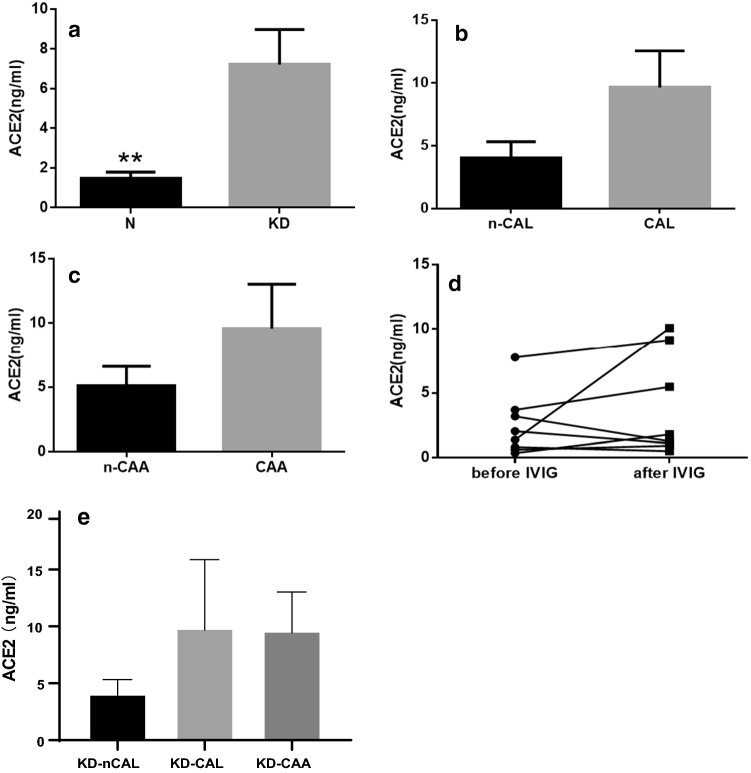
**a** Comparison of ACE2 level between KD and control groups (*p* = 0.001). **b** Comparison of ACE2 levels between n-CAL and CAL groups in KD patients (*p* = 0.089). **c** Comparison of ACE2 levels between n-CAA and CAA groups in KD patients (*p* = 0.171). **d** Comparison of ACE2 levels after IVIG therapy (*p* = 0.294). **e** Comparison of the ACE2 levels between n-CAL, CAL (excluded CAA group) and CAA groups (*p* = 0.19). Abbreviations: *ACE2*, angiotensin-converting enzyme 2; *CAL*, coronary artery lesion; *IVIG*, intravenous immunoglobulin

Serum ACE2 levels of all participants were between 0.03 and 67.44 pg/ml. As shown in Fig. 1a, the serum ACE2 concentration of the KD group was significantly higher than that of the control group (6.56 ± 1.52 vs. 1.21 ± 0.28 pg/ml) (*p* = 0.001). Figure 1b shows that ACE2 levels were higher in the KD-CAL group (9.65 ± 2.91 pg/ml) than in the KD-nCAL group (4.05 ± 1.28 pg/ml), but this difference was not statistically significant (*p* = 0.089). Figure 1c shows that ACE2 levels were higher in the KD-CAA group (9.58 ± 3.43 pg/ml) than in the KD-nCAA group (5.10 ± 1.51 pg/ml) although the difference was also not statistically significant (*p* = 0.171). As shown in Fig. 1d ACE2 content after IVIG (2.48 ± 2.46 pg/ml) was lower than prior to IVIG (3.79 ± 3.92 pg/ml), however, the difference was not statistically significant (*p* = 0.294). Figure 1e shows no difference in ACE2 levels between n-CAL, CAL (excluding CAA group) and CAA groups (4.05 ± 1.28 vs. 9.84 ± 6.04 vs. 9.58 ± 3.44, respectively, *p* = 0.19).

### Correlation between ACE2 levels and clinical parameters in patients with KD

According to the analysis of clinical data, as shown in Table 1, the levels of CK-MB in the KD-CAL group were significantly higher than those in the nCAL group; however, other clinical data were not statistically different between the two groups. There was no significant correlation between ACE2 serum levels and white blood cells, red blood cells, hemoglobin, platelets, CRP, ALT, AST, L, N, LDH, or CK-MB (*p* > 0.05); however, serum ACE2 levels were negatively correlated with Plt levels in patients with KD (*p* < 0.05) (Table 2).

**Table 1:** Clinical parameters in the KD-CAL and KD-NCAL groups

	KD-CAL	KD-nCAL	*p*
WBC (10^3^/ul)	15.16 ± 4.71	14.25 ± 6.28	0.578
RBC (10^6^/ul)	4.00 ± 0.44	3.99 ± 0.36	0.928
Hb (g/L)	105.50 ± 12.14	108.93 ± 8.95	0.262
CRP (mg/dL)	54.20 ± 36.90	73.33 ± 66.92	0.247
N (10^3^/ul)	11.16 ± 5.01	10.04 ± 4.74	0.521
L (10^3^/ul)	2.89 ± 1.86	5.50 ± 7.18	0.210
Plt (10^3^/ul)	399.05 ± 161.40	370.81 ± 107.92	0.468
ALT (U/L)	55.16 ± 53.43	53.66 ± 84.28	0.943
AST (U/L)	39.58 ± 25.64	42.26 ± 41.65	0.793
LDH (U/L)	84.82 ± 66.50	57.59 ± 64.15	0.281
CK-MB (U/L)	0.92 ± 0.95	0.20 ± 0.22	0.027*
PCT (ng/ml)	5.15 ± 8.10	1.39 ± 2.08	0.052
ESR (mm/h)	70.86 ± 23.89	72.76 ± 29.62	0.812

*WBC* White blood cell, *RBC* Red blood cell, *Hb* Hemoglobin, *CRP* C-reactive protein, *L* lymphocyte count, *N* Neutrophil count, *Plt* Platelet count, *ALT* Alanine transferase, *AST* Aspartate aminotransferase, *LDH* Lactate dehydrogenase, *CK-MB* Creatine kinase-MB, *PCT* Procalcitonin, *ESR* Erythrocyte sedimentation rate

* indicates *p* < 0.05

**Table 2:** Correlation between ACE2 levels and clinical examination parameters in patients with KD

	ACE2	
	*r*	*p*
WBC (10^3^/uL)	− 0.252	0.081
RBC (10^6^/uL)	− 0.064	0.693
Hb (g/L)	− 0.036	0.805
Plt (10^3^/uL)	− 0.307	0.032*
CRP (mg/dL)	− 0.109	0.466
ESR (mm/h)	0.103	0.492
ALT (U/L)	− 0.068	0.642
AST (U/L)	− 0.049	0.739
L (10^3^/uL)	− 0.093	0.603
N (10^3^/uL)	− 0.303	0.086
CK-MB (U/L)	− 0.100	0.502
PCT (ng/ml)	− 0.06	0.715
LDH (U/L)	− 0.002	0.991

*WBC* White blood cell, *RBC* Red blood cell, *Hb* Hemoglobin, *Plt* Platelet count *CRP* C-reactive protein, *ESR* Erythrocyte sedimentation rate, *ALT* Alanine transferase, *AST* Aspartate aminotransferase, *L* Lymphocyte count, *N* Neutrophil count, *CK-MB* Creatine kinase-MB, *PCT* Procalcitonin, *LDH* Lactate dehydrogenase

* indicates *p* < 0.05

## Discussion

ACE2 has been the first congenic compound of an angiotensin-converting enzyme discovered in recent years [14]. It is an important member of the renin-angiotensin system, which plays an important pathological role in cardiovascular and cerebrovascular diseases [15–17]. It is mainly expressed in the cardiovascular system and can convert angiotensin II to angiotensin 1–7 [18, 19]; reduce macrophage infiltration; reduce monocyte MCP-1, IL-6, TNF-α, nuclear factor-kappa B (NF-κB), VCAM-1, and ROS levels; inhibit apoptosis; and increase NO release. It protects the endothelial cells, prevents the development of atherosclerotic plaques in vivo [20], and can regulate vascular function by regulating the release of nitric oxide and oxidative stress [6, 21]. Several studies have demonstrated the role of ACE2 in vascular injury. However, the potential role of ACE2 in KD has not yet been reported. Our results showed that serum ACE2 levels in patients with KD were significantly higher than in the controls. Serum ACE2 levels in patients with KD were also positively correlated with CK-MB.

ACE2 is mainly present on the vascular endothelium, and in children with KD, it is also shed from the vascular endothelium into the serum, which in turn causes an increase in ACE2 in the serum. This finding is similar to that of ACE2 in coronary atherosclerosis. Hypertensive heart disease [5, 22, 23] can induce endothelial cell apoptosis [20] and activate NF-κB through myeloid differentiation gene response 88-dependent and -independent pathways by upregulating the expression of Toll-like receptor 4 (TLR4) on the surface of dendritic cells, initiating inflammatory cytokine transcription, mediating inflammatory mediator secretion, and producing oxygen-free radicals production [24]. The mRNA expression of TLR4 and the levels of its related factors in children in the acute phase of KD are significantly higher than those in normal children [25]. In this study, the level of ACE2 in the serum of children with KD was significantly increased, which indicated that the up-regulation of serum ACE2 may be the mechanism of vascular injury and inflammation in acute phase KD. In addition, the levels of ACE2 in KD-CAL and KD-CAA were higher than that in the control group; however, this difference was not statistically significant, which may have been related to the small number of samples or the expression of ACE2 on a variety of cells. One recent study [26] has demonstrated that ACE2 is mainly expressed in the endothelial tissues of arteries, arterioles, heart, and kidney, and is also found on the vascular smooth muscle of tubular epithelial cells, intrarenal arteries, and coronary vessels. This indicates that ACE2 may be insensitive and specific to the formation of CAL in KD patients; however, ACE2 may lead to the formation of CAL, and our study found that the level of ACE2 in KD-CAA was also increased. Its increasing trend was more obvious than that in the CAL group, which may be because vascular injury and aneurysm formation in KD were caused by inflammatory factors and oxidative stress. ACE2 has antioxidant effects in most vascular injuries [6, 27]; Therefore, we speculate that the negative results of this study may be related to the small sample size. Furthermore, KD may cause several other cardiac complications other than coronary artery injury, such as myocarditis, KD shock syndrome, valvular abnormalities, and endothelial dysfunction [28]. Whether the level of ACE2 is correlated with other complications of KD has not yet been discussed. In this study, correlation analysis showed a negative correlation between serum ACE2 and platelet levels in patients with KD. In general, the platelet count of children with KD gradually increased from the first week of onset, peaked in the 2nd–3rd week of onset, and then gradually decreased. Platelet activation level was enhanced and closely related to cardiovascular injury and mortality. The increase in platelet activation level may be involved in coronary artery injury in children with KD, and 1–2% of children with KD had decreased platelets after onset, suggesting that those with thrombocytopenia were prone to CAA [29]. In children with KD, ACE2 on the vascular endothelium sheds into the serum and loses the effect of antagonizing platelets, further leading to platelet aggregation on the vascular endothelium. This in turn induces the formation of thrombosis on the vascular endothelium, which leads to vasodilatation and vascular endothelial injury, indicating that ACE2 may be related to vascular endothelial injury and the formation of hemangioma.

In addition, our study found that the CK-MB levels in the KD-CAL group were significantly higher than those in the KD-nCAL group. CK-MB is a marker for the clinical judgment of myocarditis and myocardial injury; its increase is associated with the formation of coronary artery aneurysms [30, 31]. However, the level of ACE2 was also significantly increased in the KD-CAL group, suggesting that ACE2 may lead to the formation of CAL, and further cause vascular endothelial injury. However, our study has not found a specific link between ACE2 and CK-MB in the KD-CAL group, which needs to be confirmed in further studies. N terminal pro-B-type natriuretic peptide (NT-proBNP) is a cardiac biomarker. Several studies have found NT-proBNP to be a useful marker for diagnosis as well as for assessment of disease severity in KD [32, 33]. Yet, the NT-proBNP values of all experimental groups were not completely recorded in this study and the association between the levels of NT-proBNP and ACE2 in the pathogenesis of KD could not be further evaluated, which will be investigated in the following studies.

In conclusion, this study provides the first evidence that serum ACE2 levels are significantly increased and correlated with platelet count in patients with KD. These results suggest that high ACE2 levels may play a role in the inflammatory response and vascular injury in the acute phase of KD. However, whether and how ACE2 can cause CAL, and the association of ACE2 levels with other cardiovascular abnormalities of KD needs to be further studied.
